# α-Mangostin Induces Apoptosis and Suppresses Differentiation of 3T3-L1 Cells via Inhibiting Fatty Acid Synthase

**DOI:** 10.1371/journal.pone.0033376

**Published:** 2012-03-09

**Authors:** Xiaofang Quan, Yi Wang, Xiaofeng Ma, Yan Liang, Weixi Tian, Qingyun Ma, Hezhong Jiang, Youxing Zhao

**Affiliations:** 1 College of Life Sciences, Graduate University of Chinese Academy of Sciences, Beijing, China; 2 Institute of Tropical Bioscience and Biotechnology, Chinese Academy of Tropical Agricultural Sciences, Haikou, China; 3 State Key Laboratory of Phytochemistry and Plant Resources in West China, Kunming Institute of Botany, Chinese Academy of Sciences, Kunming, China; National Institute of Agronomic Research, France

## Abstract

α-Mangostin, isolated from the hulls of *Garcinia mangostana* L., was found to have *in vitro* cytotoxicity against 3T3-L1 cells as well as inhibiting fatty acid synthase (FAS, EC 2.3.1.85). Our studies showed that the cytotoxicity of α-mangostin with IC_50_ value of 20 µM was incomplicated in apoptotic events including increase of cell membrane permeability, nuclear chromatin condensation and mitochondrial membrane potential (ΔΨm) loss. This cytotoxicity was accompanied by the reduction of FAS activity in cells and could be rescued by 50 µM or 100 µM exogenous palmitic acids, which suggested that the apoptosis of 3T3-L1 preadipocytes induced by α-mangostin was via inhibition of FAS. Futhermore, α-mangostin could suppress intracellular lipid accumulation in the differentiating adipocytes and stimulated lipolysis in mature adipocytes, which was also related to its inhibition of FAS. In addition, 3T3-L1 preadipocytes were more susceptible to the cytotoxic effect of α-mangostin than mature adipocytes. Further studies showed that α-mangostin inhibited FAS probably by stronger action on the ketoacyl synthase domain and weaker action on the acetyl/malonyl transferase domain. These findings suggested that α-mangostin might be useful for preventing or treating obesity.

## Introduction

Obesity is a complex metabolic disorder, which affects normal functions of the whole body. Since involved in various serious diseases including type 2 diabetes, hypertension, coronary heart disease, apoplexy, osteoarthritis and cancers, obesity has become a worldwide public health threat [1]. Many parts of human body can be served as targets in treating obesity, one of which is aim at fat storage tissue by regulating fat synthesis/lipolysis/adipose differentiation or apoptosis [2]. Fatty acid is an important source of fat synthesis, but excess of their ectopic accumulation in other functional organs will lead to lipotoxicity, fatty liver and insulin resistance or other obesity-related diseases [3]. The *de novo* synthesis of long chain fatty acids are catalyzed by fatty acid synthase (FAS, EC 2.3.1.85), which has been considered as an anti-obesity target recently. FAS not only connects with metabolic substrates, and represents an important link in feeding regulation [4]–[6]. C75, a traditional FAS inhibitor, could inhibit orexis in the central system and stimulate carnitine palmitoyltransferase-1 (CPT-1), which promotes the oxidation of fatty acids and increases the levels of ATP in the periphery [6], [7]. Therefore, inhibiting FAS may significantly reduce weight and treat obesity under the dual mechanism [8].

Obesity is caused by increased adipose tissue mass, which resulting from increased fat-cell numbers (hyperplasia) and size (hypertrophy), accompanies by the unbalance between energy intake and expenditure [9]. Adipose tissue consists of mature adipocytes, pradipocytes, endothelial cells, macrophages, fibroblasts, and adiposederived stem cells (ADSC), among which approximately one third is mature adipocytes and the remaining is a combination of small blood vessels, nerve tissue, fibroblasts and preadipocytes in various stages of development [10]. Preadipocytes are capable to propagate and differentiate into mature adipocytes, which determines the number of fat cells throughout their entire lifespan [11]. Meanwhile, the size of fat-cell depends on the lipids accumulation in the adipocytes. Therefore, adipose tissue mass can be reduced by the inhibition of adipogenesis from preadipocytes to mature adipocytes, prevention of lipid accumulation in adipocytes, and induction of apoptosis in adipose cells, which can also contribute to the treatment of obesity.

The fruit hulls of *Garcinia mangostana* Linn, family Guttiferae, has been used for hundreds of years around the world, mainly in Southeast Asia, as a traditional herbal medicine for the treatment of abdominal pain, dysentery, wound infections, eczema, suppuration, and chronic ulcer [12], [13]. α-Mangostin, the dominant xanthone found from the fruit hulls of *Garcinia mangostana* L., has been demonstrated by pharmacological studies to possess antioxidant [14]–[16], antibacterial [12], [17], [18], antiinflammatory [19], antitumor [20]–[29] and renoprotective [26] activities.

This study investigated for the first time the effect of α-mangostin, isolated from the hulls of *G. mangostana* L., to FAS, and the subsequent apoptotic effect on 3T3-L1 preadipocytes, promotion of mature adipocytes lipolysis and inhibition of lipid accumulation during the differentiation of 3T3-L1 preadipocytes into mature adipocytes. And explore the utilite prospective of α-mangostin as a drug candidate in treating obesity.

## Results

### Inhihitory effect of α-mangostin on viability of 3T3-L1 preadipocytes

**Figure 1:**
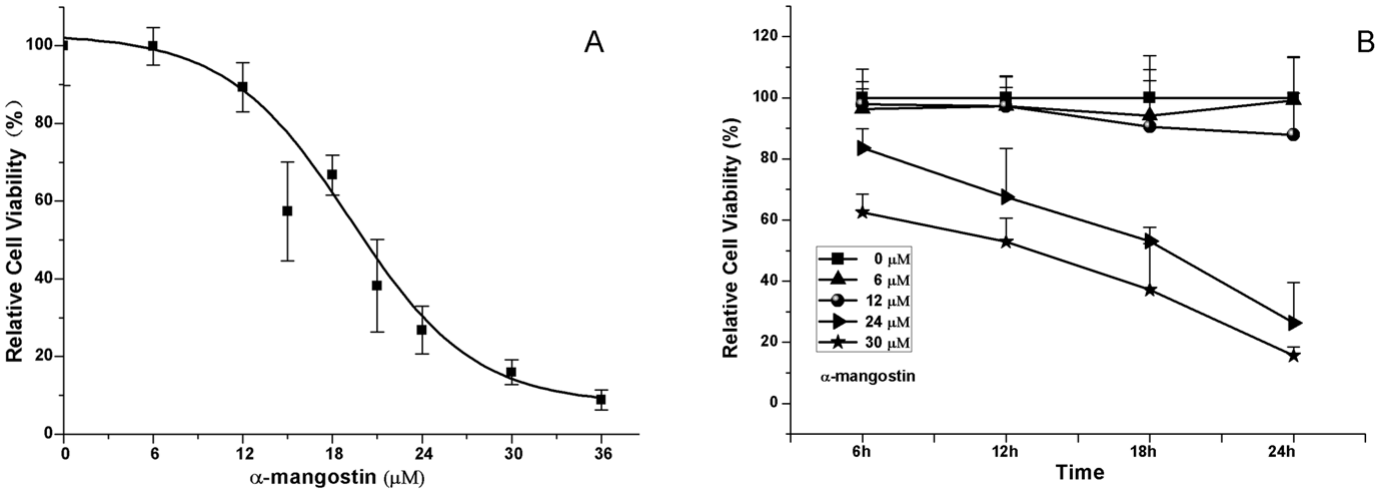
Effect of α-mangostin on proliferation of 3T3-L1 preadipocytes. (A) Cells were incubated with 0–36 µM α-mangostin for 24 h at 37°C in humidified 5% CO_2_ incubator. (B) Cells were incubated with 0, 6, 12, 24, 30 µM α-mangostin for 6–24 h at 37°C in humidified 5% CO_2_ incubator. Assays were performed on eight replicates for each treatment. Results are expressed as percentages of cell viability as compared with untreated control (means ± S.D., n = 8). The experiments were repeated in twice.

To identify whether α-mangostin could inhibit the proliferation of 3T3-L1 preadipocytes, the cells were treated with 0–36 µM α-mangostin and proliferative capability was determined by MTT assay. As shown in Fig. 1, α-mangostin showed strong inhibition on cell population growth in a dose- and time-dependent manner with 50% growth inhibitory concentration (IC_50_) value of 20 µM, and it needed 13.5 h to inhibit 50% cell population growth while in the concentration of 30 µM .

### Apoptotic effect of α-mangostin on 3T3-L1 preadipocytes

**Figure 2:**
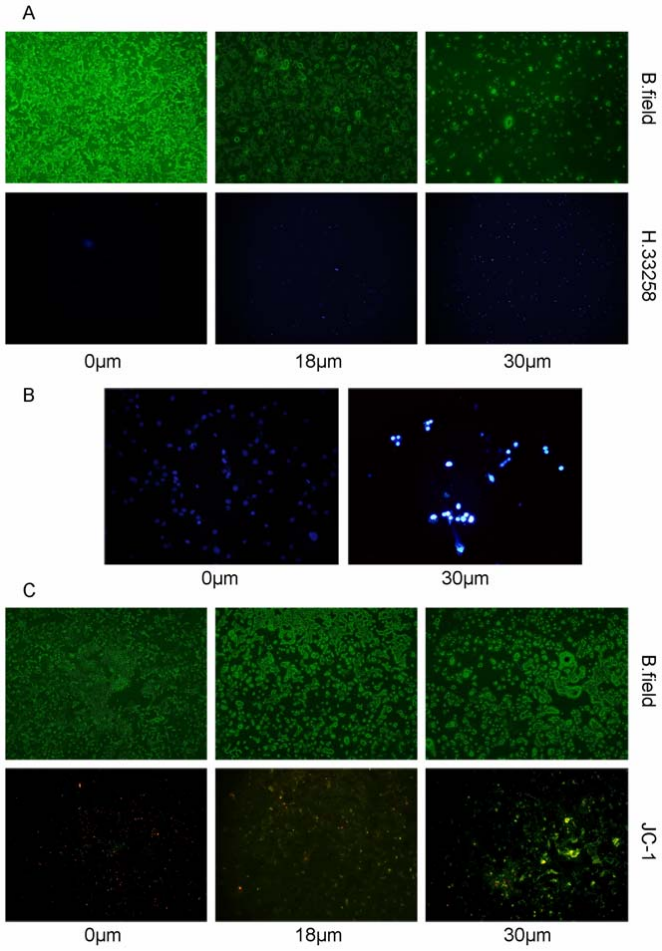
α-mangostin-induced 3T3-L1 preadipocytes apopotosis. 3T3-L1 preadipocytes were treated with α-mangostin at the indicated concentrations for 24 h. (A) Effect of α-mangostin on cell membrane permeability: original magnification, ×40; exposure times: 20s; (B) Effect of α-mangostin on nuclear chromatin morphology with Hoechst 33258 staining: original magnification, ×200; exposure times: 100s. (C) Effect of α-mangostin on mitochondria membrane potential (ΔΨm) with JC-1 staining: original magnification, ×40; exposure times: 100 s. B. field, bright field; H.33258, Hoechst 33258; JC-1, 5,5,6,6-tetrachloro-1,1,3,3-tetraethylbenzimidazolcarbocyanine iodide. The experiments were performed on four replicates for each treatment. Representative images are shown.

In order to examine whether the inhibitory effect on 3T3-L1 preadipocytes by α-mangostin was due to it induced apoptotic cell death, several apoptotic events including increase of cell membrane permeability, nuclear chromatin condensation and mitochondrial membrane potential (ΔΨm) loss were tested. After exposed to 0.1% DMSO (control) or α-mangostin (18 µM and 30 µM) for 24 h, apoptosis of 3T3-L1 preadipocytes was demonstrated by Hoechst 33258 staining, revealed cell membrane permeability increasement and nuclear condensation (Fig. 2A & B), and by JC-1 staining, which showed the fluorescence changed from red to green, reflected the collapse of mitochondria membrane potential (Fig. 2C).

### The effects of α-mangostin on 3T3-L1 preadipocytes is related to its inhibition to FAS

**Figure 3:**
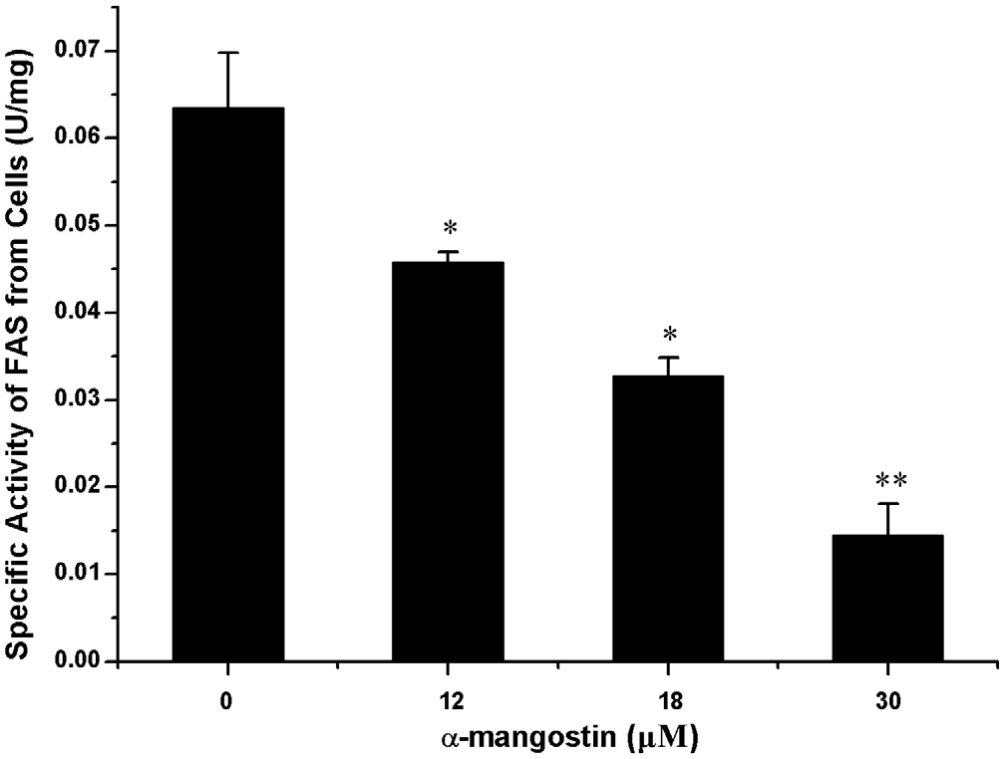
Effect of α-mangostin on FAS activity in 3T3-L1 preadipocytes. 3T3-L1 preadipocytes were treated with α-mangostin at the indicated concentrations for 24 h. FAS specific activity was determined by *Cell FAS activity assay*. Data are expressed as means ± S.D. (*n* = 3). * *p*<0.05 different from control (0 µM); ** *p*<0.01significantly different from control (0 µM).

Since α-mangostin was found to have inhibitory activity on FAS, we extended our investigation to the intracellular FAS activities after treated with α-mangostin. As shown in Fig. 3, 3T3-L1 preadipocytes treated with α-mangostin at the concentration of 12 µM, 18 µM and 30 µM for 24 h, after which the reduction of intracellular FAS activities at a level of 30%, 50% and 80%, compared with control.

**Figure 4:**
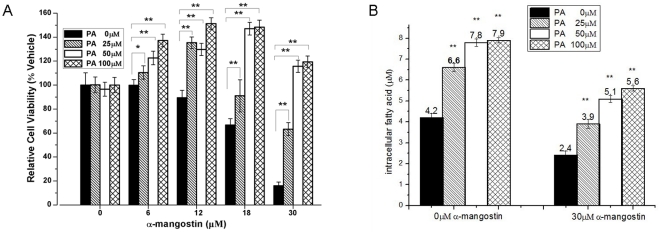
The effect of exogenous palmitic acid on 3T3-L1 preadipocytes. (A) 3T3-L1 preadipocytes were treated with α-mangostin and palmitic acid at various concentrations (α-mangostin: 0, 6, 12, 18, 30 µM; palmitic acid: 0, 25, 50, 100 µM) for 24 h. Cell viability was determined by MTT colorimetric assay. Assays were performed on eight replicates for each treatment. Results are expressed as percentages of cell viability as compared with untreated control (means ± S.D., n = 8). The experiment was repeated in twice. (B) The 3T3-L1 preadipocytes were treated with α-mangostin and palmitic acid at various concentrations (α-mangostin: 0, 30 µM; palmitic acid: 0, 25, 50, 100 µM) for 24 h. And then the amount of intracellular fatty acid was determined by Fatty Acid Assay Kit. Data are expressed as means ± S.D. (*n* = 3). * *p*<0.05 different from respective control; ** *p*<0.01 significantly different from respective control.

To confirm that the apoptotic effect induced by α-mangostin was related to FAS inhibition, 3T3-L1 preadipocytes were exposed to 0–30 µM α-mangostin for 24 h in the presence of 0, 25, 50, 100 µM palmitic acid. Results showed that 50 µM and 100 µM exogenous palmitic acid could completely rescue the apoptosis induced by α-mangostin (Fig. 4A). In addition, the amount of intracellular fatty acids in 3T3-L1 preadipocytes, treated or untreated with 30 µM α-mangostin in the presence of additional different concentrations of palmitic acids or not, were measured. Results (Fig. 4B) showed that the intracellular level of fatty acids in treated 3T3-L1 preadipocytes with 30 µM α-mangostin decreased by 43% compared with the control (4.2 µM). When 25 µM, 50 µM and 100 µM additional exogenous palmitic acids were added into cell culture media, the amount of intracellular fatty acids in 3T3-L1 preadipocytes raised to 3.8 µM, 5.1 µM and 5.6 µM. However, exogenous palmitic acids seem to have no effect on α-mangostin-untreated 3T3-L1 preadipocytes, though the amount of intracellular fatty acids increased.

These results suggest that the effect of α-mangostin on 3T3-L1 preadipocytes is well correlated with its inhibitory activity on FAS.

### Effects of α-mangostin on the lipogenesis and lipolysis of 3T3-L1 preadipocytes

**Figure 5:**
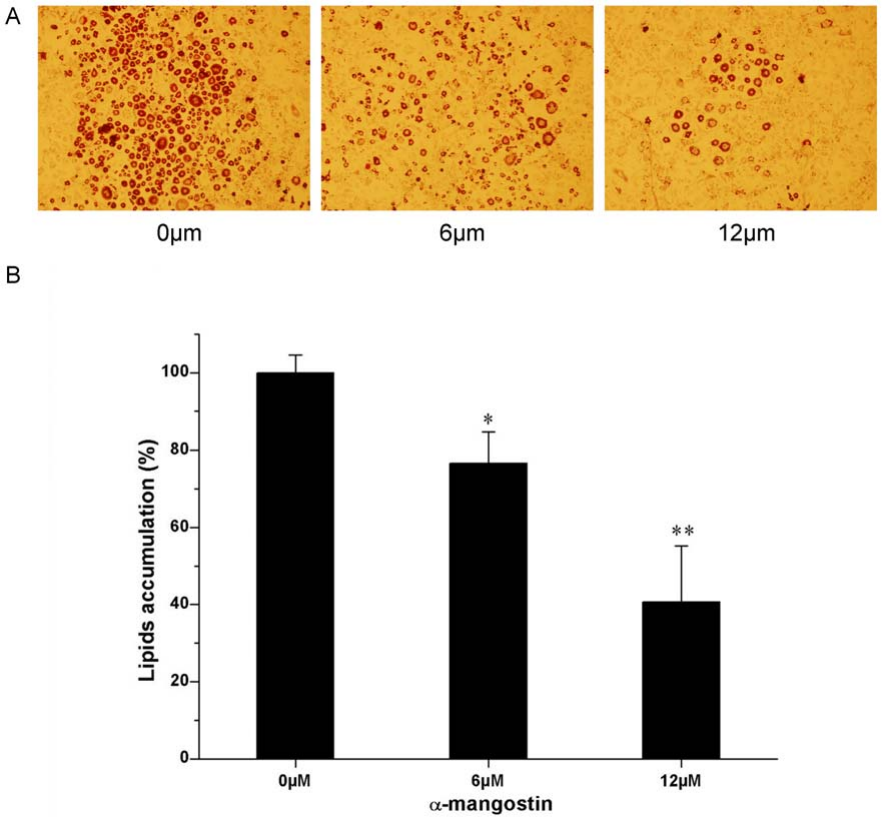
Inhibitory effect of α-mangostin on intracellular lipid accumulation. The intracellular lipid content was measured by *Oil Red O staining* as described in Materials and Methods. (A) Cells were photographed at 40× magnification. The experiment was performed on three replicates for each treatment. Representative images are shown. (B) Quantitative analysis of lipid accumulation. Each value is expressed as means ± SD (*n* = 3). * *p*<0.05 different from control (0 µM); ** *p*<0.01 significantly different from control (0 µM).

**Figure 6:**
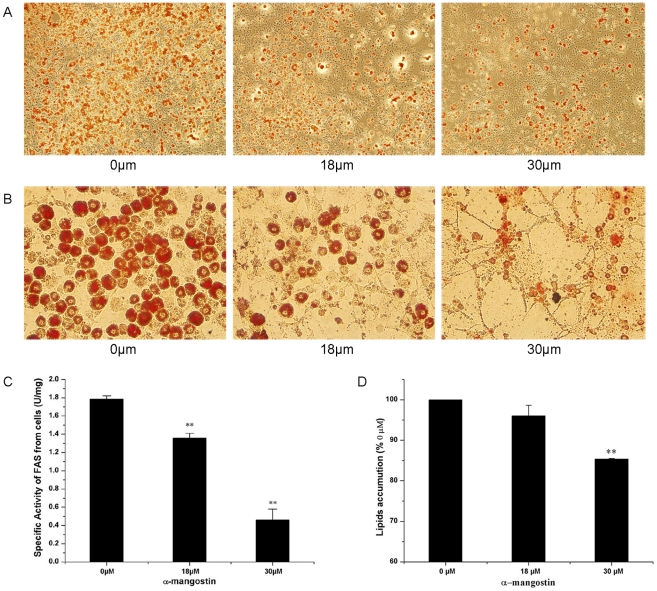
Effect of α-mangostin on lipolysis and FAS activity in 3T3-L1 mature adipocytes. 3T3-L1 mature adipocytes were treated for at the indicated concentrations for 24 h, and then the intracellular lipid content, cell FAS activity were measured and cell sizes were compared. (A) Cells were photographed at 40× magnification. The experiment was performed on three replicates for each treatment. Representative images are shown. (B) Cells were photographed at 200× magnification. The experiment was performed on three replicates for each treatment. (C) Reduction of FAS activity. Data are expressed as means ± S.D. (*n* = 3).(D) Quantitative analysis of lipid lipolysis. Each value is expressed as means ± SD (*n* = 3). * *p*<0.05 different from control (0 µM); ** *p*<0.01 significantly different from control (0 µM).

To examine whether α-mangostin could suppress the differentiation of 3T3-L1 preadipocytes at a low dosage without cytotoxicity, the cells were exposed continuously to 6 and 12 µM α-mangostin during the differentiation period for 8 days, and then the intracellular lipid accumulation was determined. As shown in Fig. 5, 6 and 12 µM α-mangostin failed to affect the cell viability, but reduced the lipid accumulation by 20% and 60%, compared with control, respectively. For mature 3T3-L1 adipocytes, cell sizes were shrunk when treated with 18 and 30 µM α-mangostin (Fig. 6B). In addition, 30 µM α-mangostin promoted 15% lipid to lipolysis, compared to control (Fig. 6D). Simultaneously, 74% FAS activity was inhibited (Fig. 6C).

### Effect of α-mangostin on viability of 3T3-L1 preadipocytes in different differentiationstages

**Figure 7:**
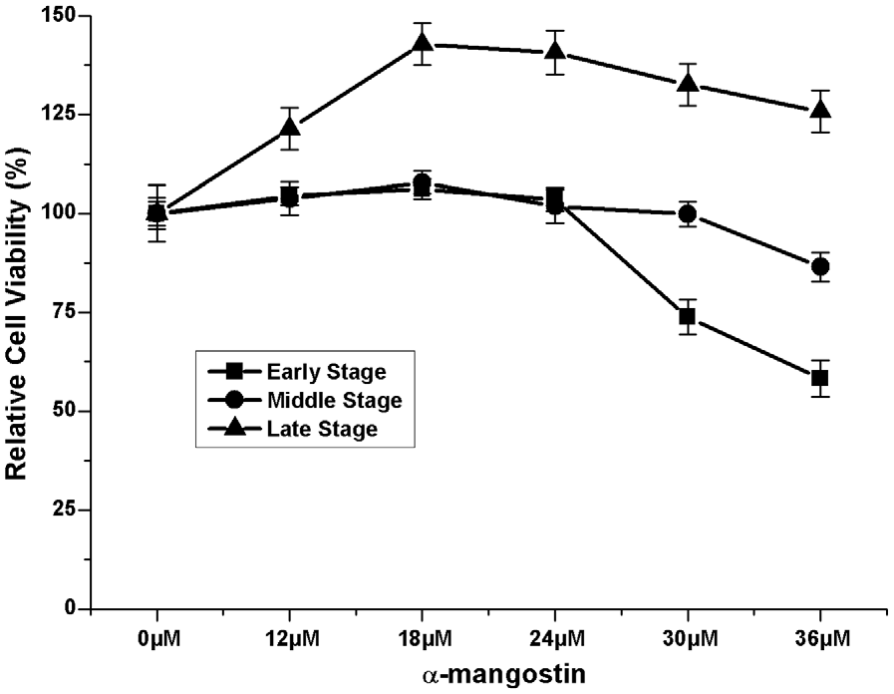
Effect of α-mangostin on cell viability of 3T3-L1 cells in different stages of adipocyte differentiation. 3T3-L1 cells were exposed to various concentrations of α-mangostin at various time points (d_2_, d_4_ or d_8_) for 24 h, then the cell viability were measured by MTT assay. Assays were performed on four replicates for each treatment. Results are expressed as percentages of cell viability as compared with each untreated control (means ± S.D., *n* = 4). The experiment was repeated in triplicate. Early stage: d_0_-d_2_; Middle stage: d_2_-d_4_; Late stage: d_4_-d_8_.

To examine further effect of α-mangostin on different stages of adipocyte differentiation, 3T3-L1 cells were exposed to a series concentrations of α-mangostin for 24 h at given time points (d_2_, d_4_ or d_8_), then the cell viability were measured (Fig. 7). Interestingly, as 3T3-L1 preadipocytes differentiated, they appeared to gain the ability to resist the suppression from α-mangostin. As shown in Fig. 1, 50% 3T3-L1 preadipocytes lost their cell viability when treated with 20 µM α-mangostin. However, after cells were induced for 4 days, only 10% cells lost their viability when treated with 36 µM α-mangostin. After 3T3-L1 cells developed into mature adipocytes, 36 µM α-mangostin had no cytotoxic effect on cells. These results indicated that 3T3-L1 preadipocytes were more susceptible to the cytotoxic effect of α-mangostin than mature adipocytes.

### Kinetic mechanism of inhibition of FAS by α-mangostin

**Figure 8:**
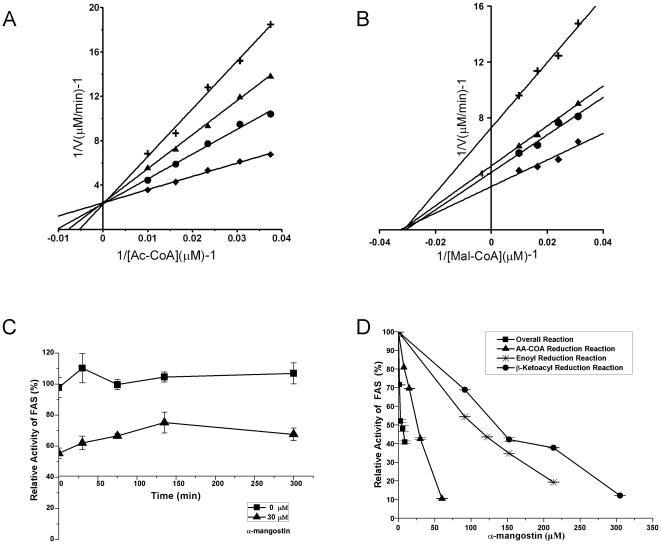
Inhibitory effects of α-mangostin on FAS activities. (A) and (B) Lineweaver-Burk plots for inhibition of FAS overall reaction: (A) Acetyl-CoA was the variable substrate. The concentrations of α-mangostin were 0 µM (⧫); 5 µM (•); 10 µM (▴); 15 µM (**+**). (B) Malonyl-CoA was the variable substrate. The concentrations of α-mangostin were 0 µM (⧫); 5 µM (•); 10 µM (▴); 15 µM (**+**). (C) Time courses of the overall reaction in the presence of α-mangostin. The reaction was determined by slow-binding inhibition assay. (D) Inhibition of FAS overall reaction and some partial reactions of FAS.

In order to explore the inhibition mechanism on FAS of α-mangostin, the inhibitory kinetics was studied. Lineweaver-Burk analysis of the kinetic data showed that α-mangostin inhibited the overall reaction of FAS in a competitive manner with respect to acetyl-CoA (Fig. 8A) and a noncompetitive manner with respect to malonyl-CoA (Fig. 8B). α-Mangostin had no slow-binding irreversible inhibition effect on FAS (Fig. 8C).

These results suggest that α-mangostin might act reversibly on the acetyl binding group of FAS but not malonyl group. So the acting sites of α-mangostin on FAS were most likely on acetyl/malonyl transferase domain (MAT), or β-ketoacyl synthase domain (KS) or both. The activities of individual reactions in FAS overall reactions were measured in the presence of α-mangostin (Fig. 8D). It is found that β-ketoacyl and enoyl reduction reactions were slightly influenced by α-mangostin, which indicates that α-mangostin does not act on the β-ketoacyl reductase (KR) and enoyl reductase (ER) domains of FAS. The results are consistent with former hypothesis. In order to define the actual acting sites of α-mangostin on FAS, we assayed the effect of α-mangostin against acetoacetyl-CoA reduction which entails catalytic reaction by MAT. As shown in Fig. 1D, α-mangostin has the ability to inhibit acetoacetyl-CoA reduction of FAS, with IC_50_ value of 28 µM, which is about 5-fold weaker than that of FAS overall reaction (IC_50_ = 5.54 µM). This comparison shows that α-mangostin strongly acts on the domain of FAS which is not included in the domains required in the acetoacetyl-CoA reduction. So we conclude that α-mangostin inhibits FAS on the acetyl binding sites of KS more stronger than that of MAT.

## Discussion

Inhibition of FAS in animal reduced food intake and body weight, which suggested that inhibiting intracellular FAS should be a reasonable way for treating obesity and related diseases. As a novel FAS inhibitor, α-mangostin may be applied practically in treating obesity or health care. The present study showed that α-mangostin can reduce adipose tissue mass by reducing fat-cell numbers and shrinking their sizes (See Fig. 5 and Fig. 6A, 6B).

Firstly, when 3T3-L1 preadipocytes were treated with α-mangostin, their viabilities appeared to be reduced in a dose- and time-dependent manner, with the IC_50_ value of 20 µM. About 90% of 3T3-L1 preadipocytes lost their viabilities by treating with 36 µM α-mangostin in 24 h. However, 6–12 µM α-mangostin had slightly cytotoxicity to preadipocytes (Fig. 1). Higher concentration of α-mangostin could induce cell apoptosis of 3T3-L1 preadipocytes which was evidenced by increased cell membrane permeability, nuclear chromatin condensation and mitochondrial membrane potential (ΔΨm) loss (Fig. 2). Induction of apoptosis in cells can lead to their own destruction into apoptotic bodies which can be cleared by surrounding phagocytic cells without inducing a local damaging inflammatory response [30], [31], so apoptosis is thought to be a normal and efficient way by which human body weeds out dead and waste cells. Like other cells, adipose cell has its own lifespan, during which preadipocytes propagate and differentiate into mature adipocytes to renew, replenish and keep the numbers of fat cells. Since α-mangostin can reduce preadipocytes numbers, and accordingly cause the reduction of adipose cells, adipose tissue treated with α-mangostin avoids hyperplasia.

Secondly, lower concentration of α-mangostin (6 µM and 12 µM) inhibits lipid accumulation during adipose differentiation period. As shown in Fig. 5, although 6 µM and 12 µM α-mangostin failed to induce cell apoptosis, they reduce the lipid accumulation by 20% and 60% of control, respectively. It indicated that the reduction of lipid resulted from inhibition of lipid accumulation, but not from reducing number of preadipocytes. Also, α-mangostin in higher concentration promotes lipid to lipolysis but not decrease cell numbers for the muture adipocytes seemed to gain the ability to resist the suppressive effect of α-mangostin. Therefore, inhibition of adipogenesis and stimulation of lipolysis by α-mangostin may be a beneficial method to control hypertrophy in adipose tissue. Recent studies have shown that many FAS inhibitors could suppress lipid accumulation during adipocyte differentiation [32]–[35]. The effect of α-mangostin on 3T3-L1 preadipocytes and muture adipocytes, as shown in Fig. 3 and Fig. 6C, is also according to the inhibition of FAS activity. It shows that α-mangostin can decrease the amount of intracellular fatty acids in 3T3-L1 preadipocytes, but additional exogenous palmitic acids can raise the amount of intracellular fatty acids (Fig. 4B). And the cytotoxicity of α-mangostin on 3T3-L1 preadipocytes can also be rescued by 50 µM and 100 µM exogenous palmitic acid (Fig. 4A). It is indicated that the apoptotic effect of α-mangostin on 3T3-L1 cells is related to its inhibition on FAS. Inhibition of FAS has been reported to promote apoptosis by activating the cellular intrinsic pathway of apoptosis, which was demonstrated by annexin V-positive cells, the cytochrome c release and caspase-9 and -3 activation [36]. In addition, inhibition of FAS results in the accumulation of malonyl-CoA, which leads to inhibition of CPT-1 and up-regulation of ceramide and induction of the proapoptotic genes BNIP3, TRAIL, and DAPK2, resulting in apoptosis [37].

In our previous study, α-mangostin was found to inhibit FAS overall reaction with IC_50_ value of 5.54 µM [38]. In this study, we assayed its inhibition kinetics to explore its specific acting sites on FAS. After pertinent experiment, we found that the β-ketoacyl and enoyl reductions of FAS were slightly influenced by α-mangostin, which indicates that the two reductases domains are not the acting site of α-mangostin (Fig. 8D). The inhibition kinetic results *in vitro* showed that α-mangostin inhibited FAS against substrate acetyl CoA competitively, and against another substrate malonyl CoA noncompetitively (Fig. 8A–B), which suggested that α-mangostin may bind to acetyl but not malonyl group on FAS. In FAS, KS could bind to acetyl but not malonyl group [39], [40], and MAT has different active binding sites for acetyl and malonyl [41]. Therefore, KS and MAT are likely to be the sites α-mangostin may act on. To figure out clearly which sites α-mangostin may act on, acetoacetyl CoA reduction activity, which includes catalytic reaction by MAT but not KS, was assayed. α-Mangostin inhibited this reaction with IC_50_ value of 28 µM (Fig. 8D) that is about 5-fold of that of the inhibition for FAS overall reaction. These results suggested that α-mangostin inhibit FAS probably by stronger action on the KS domain and weaker action on the MAT domain.

In conclusion, our study indicates that α-mangostin inhibits intracellular FAS by mainly acting on the KS domain of FAS. Moreover, α-mangostin is effective in inducing apoptosis of preadipocyte, suppressing adipocyte differentiation, reducing lipid accumulation and causing lipolysis of mature adipocyte, which is due to the inhibition of FAS. The mechanism of this result is unclear. It has been shown that the specific activity of FAS in mature adipose cells is greatly higher than in preadipocytes (Fig. 3 & Fig. 6). Although 30 µM α-mangostin could suppress 74% FAS activity (Fig. 6), it is not enough to reach the degree to induce cell apoptosis. So it is likely to need higher concentration of α-mangostin to reach the same suppressive effect with it testing on undifferentiated cells. Another explanation is that during adipocyte differentiation, some resistance factors are expressed by induction. Level of neuronal apoptosis inhibitory protein (NAIP) was reported to augment, which is closely correlated with the development of resistance to apoptosis induced by growth factor deprivation [42]. NAIP is one of the inhibitors of apoptosis (IAPs) family, which suppress apoptosis [43]. Besides, it is found that there was a significant increase during adipocyte differentiation in the expression of Bcl-2 protein which retains cytochrome c within the mitochondria, suggesting a possible role for this pro-survival factor in the acquisition of apoptotic resistance in adipose cells [44]. Perhaps these factors also play the role in mature adipocyte cells resistance to apoptosis induced by α-mangostin.

Considering multitargeted therapy is possibly better than monotargeted therapy for obesity, our results suggest that α-mangostin could be considered to have the application potential in preventing or treating obesity, and it may supply some useful idea and new clues in developing drugs in treatment of obesity.

## Materials and Methods

### Materials

Acetyl-CoA, Malonyl-CoA, Insulin (INS), Dexamethasone (DEX), Oil red O, NADPH, MTT dye, Hoechst-33258, 3-isobutyl-1-methylxanthine (IBMX), Palmitic acid, EDTA and DTT were purchased from Sigma. Dulbecco's modified Eagle's medium (DMEM) and fetal bovine serum were purchased from GIBCO BRL. α-Mangostin (≥98%) was isolated and purified from the hulls of *G. mangostana* L. as described previously [38]. All other reagents were local products with analytical grade.

### Cell culture

#### Culture of 3T3-L1 preadipocytes

Mouse 3T3-L1 preadopocytes were obtained from the Cell Culture Center of the Institute of Basic Medical Sciences (IBMS), Chinese Academy of Medical Sciences (Beijing, China). 3T3-L1 preadipocytes were incubated in DMEM, 10% fetal bovine calf serum, 100 U/ml penicillin-streptomycin.

#### Differentiation of 3T3-L1 preadipocytes

Two days after confluence (Day 0, d_0_), the cell differentiation was induced in DMEM containing 10% FBS, 0.5 mM IBMX, 1 µM DEX, and 1.7 µM INS for two days (Day 2, d_2_, early stage), and another two days (Day 4, d_4_, middle stages) in DMEM containing 10% FBS and 1.7 µM INS. Cells were maintained in DMEM containing 10% FBS every other day for the following four days (Day 6–8, d_6–8_, late stage).

All experiments were strictly carried out on cell lines between 5–20 passages. All cell culture condition was 37°C in a humidified 5% CO_2_ incubator. The α-mangostin was conserved in DMSO before added into the culture medium.

### MTT assay

3T3-L1 preadipocytes were seeded in 96-well plate (5×10^3^ cells/well) and then treated with α-mangostin in different concentrations for the next 6–24 hrs. Thereafter, 20 µl of MTT solution (5 mg MTT/mL in PBS) was added into each well of a microtiter plate and incubated for 4 h at 37°C. The resultant formazan product was dissolved in 200 µl DMSO/well, and its concentration was measured at 492 nm by a microplate spectrophotometer (Multiskan, MK3).

To verify the effects of α-mangostin on 3T3-L1 cells preadipocytes at different adipogenic stage (early, middle and late stage), the cell viability was measured by the MTT assay. Briefly, 2×10^5^ cells/well were seeded in 24-well plate and α-mangostin in different concentrations was added into the wells at d_2_, d_4_, d_8_ and then incubated at 37°C for 24 h. The MTT assay was performed according to the above instructions.

### Intracellular fatty acids assay

The amount of intracellular fatty acid was determined by Fatty Acid Assay Kit. Briefly, 3T3-L1 preadipocytes were seeded in 100 mm cell culture dishes. After experimental treatment, cells were washed twice with PBS, and then extracted by homogenization with 200 µl of chloroform-Triton X-100 (1% Triton X-100 in pure chloroform) in a microhomogenizer. Then spin the extract 5–10 min at top speed in a microcentrifuge. Collect organic phase (lower phase), air dry at 50°C to remove chloroform. Vacuum dry 30 min to remove trace chloroform. Dissolve the dried lipids in 200 µl of Fatty Acid Assay Buffer by vortexing extensively for 5 min. Add 2 µl ACS Reagent into all sample wells and incubate the reaction at 37°C for 30 min. Add 50 µl of the Reaction Mix containing 44 µl Assay Buffer, 2 µl Fatty Acid Probe, 2 µl Enzyme Mix and 2 µl Enhancer to the test samples. Incubate the reaction for 30 min at 37°C, protected from light. The colorimetric assay was measured at 570 nm by a microplate spectrophotometer.

### Hoechst 33258 Staining

3T3-L1 preadipocytes were seeded in 12-well culture dishes (5×10^4^ cells/well). After experimental treatment, cells were washed twice with PBS, and stained with Hoechst-33258 (5 µg/ml) for 5 min in the dark, and then followed by extensive washes. Nuclear staining was examined under the fluorescence microscope and images were captured using ImagePro Plus software (MediaCybernetics, Silver spring, MD).

### Mitochondria Membrane Potential (ΔΨm) Assay

Mitochondria membrane potential was determined by using the mitochondrial membrane potential assay kit with JC-1 (Beyotime Biotech, Nantong, China). Briefly, 3T3-L1 preadipocytes were seeded in 12-well plates. After experimental treatment, cells were washed twice with PBS, and then added 1 ml staining dye/well (culture medium: JC-1 working dye  = 1∶1) and incubated at 37°C for 20 min. After this, cells were washed twice with cold JC-1 staining buffer (1×), and examined under the fluorescence microscope and images were captured using ImagePro Plus software (MediaCybernetics, Silver spring, MD).

### Oil Red O staining

To investigate the effects of α-mangostin on lipid accumulation in 3T3-L1 preadipocytes, the cells were differentiated in the presence of α-mangostin at various concentrations. Intracellular lipid accumulation was determined by Oil Red O staining at day 8. Cells were washed twice with PBS and stained with Oil Red O (six parts 0.6% Oil Red O dye in isopropanol and four parts water) for 1 h. After washed three times with distilled water, cells were photographed under the microscope. Lipid and Oil red O were dissolved in isopropanol and absorbance was measured by the microplate spectrophotometer (Multiskan, MK3) at the wavelength of 492 nm.

### Cell FAS activity assay

FAS activity in cells was assessed as described previously with appropriate modifications [21]. After cells were harvested, pelleted by centrifugation, resuspended in cold assay buffer (100 mM potassium phosphate buffer, 1 mM EDTA, 0.6 mM PMSF and 1 mM dithiolthreitol, pH 7.0), ultrasonically disrupted and centrifuged at 12000 rpm for 30 min at 4°C, the supernatant was collected for the overall reaction assay. 25 µl supernatant was added into the reaction mix contained 25 mM KH_2_PO_4_-K_2_HPO_4_ buffer, 0.25 mM EDTA, 0.25 mM dithiothreitol, 30 µM acetyl-CoA, 100 µM malonyl-CoA, 350 µM NADPH (pH 7.0) in a total volume of 200 µl. Protein content in the supernatant was determined using a bicinchoninic acid (BCA) assay (Pierce) and results are expressed as the specific activity of FAS (U/mg).

### FAS activity assay

#### Preparation of FAS and its substrate

The preparation, storage and use of fatty acid synthase from chicken liver were performed as described previously [45]. The amino acid sequence of human FAS has 63% identity with the sequences of chicken enzymes [46]. The purified FAS was homogeneous on polyacrylamide gel electrophoresis in the presence and absence of sodium dodecyl sulfate (SDS). The concentrations of the enzyme and substrate were determined by Amersham Pharmacia Ultrospec 4300 pro UV-Vis spectrophotometer (England, UK) spectrophotometer using the following extinction coefficients: FAS, 4.83×10^5^ M^−1^ cm^−1^ at 279 nm; acetyl-CoA, 1.54×10^4^ M^−1^ cm^−1^at 259 nm, pH 7.0; malonyl-CoA,1.46×10^4^ M^−1^ cm^−1^at 260 nm, pH 6.0; NADPH, 6.02×10^3^ M^−1^ cm^−1^ at 340 nm, and 1.59×10^4^ M^−1^ cm^−1^at 259 nm, pH 9.0 [47].

#### Assays of FAS activity

The FAS activity was measured by following the decrease of NADPH absorption at 340 nm using an Amersham Pharmacia Ultrospec 4300 pro UV-Vis spectrophotometer at the constant temperature of 37°C. The overall reaction system contained 100 mM KH_2_PO_4_-K_2_HPO_4_ buffer, 1 mM EDTA, 1 mM dithiothreitol, 3 µM acetyl-CoA, 10 µM malonyl-CoA, 35 µM NADPH, and 10 µg FAS in a total volume of 2 ml as previously described [45], [47], [48]. The acetoacetyl-CoA reduction reaction mixture contained 100 mM KH_2_PO_4_-K_2_HPO_4_ buffer, pH 7.6, 25 µM acetoacetyl-CoA, 35 µM NADPH, 1 mM EDTA and 40 µg FAS in a total volume of 2 ml. Acetoacetyl-CoA reduction reaction includes transacylation, β-ketoacyl reduction, dehydration and enoyl reduction, which are catalyzed by four component enzymes and ACP in fatty acid synthase [49]. After temperature equilibration at 37°C for 10 min, the reaction was initiated by the addition of FAS and the initial velocity was determined from the decrease in absorbance at 340 nm. β-ketoacyl reduction reaction mixture contained 100 mM KH_2_PO_4_-K_2_HPO_4_ buffer, pH 7.0, 200 mM ethyl acetoacetate, 35 µM NADPH, 1 mM EDTA and 20 µg FAS in a total volume of 2 ml [50]. The enoyl reduction reaction mixture contained 100 mM KH_2_PO_4_-K_2_HPO_4_ buffer, pH 6.3, 40 mM ethyl crotonate, 35 µM NADPH, 1 mM EDTA and 80 µg FAS in a total volume of 2 ml.

#### Assays of FAS inhibition

Fast-binding inhibition was determined by adding α-mangostin to the reaction system before FAS initiated the reaction. The activities of FAS with and without α-mangostin were represented as A_i_ and A_0_. The value of A_i_/A_0_×100% was the remaining activity (R.A.) of FAS. The value of 50% inhibition concentration (IC_50_) was calculated from the plot of remaining activity versus α-mangostin concentration with Origin 8.0 program. Dissolution of α-mangostin was lower in 0.5% (V/V) dimethyl sulfoxide (DMSO), while FAS activity was not affected by this concentration of DMSO (data not shown).

For the slow-binding inhibition assay, FAS solutions was incubated with α-mangostin at room temperature for 0–300 min, and aliquots were taken to measure the remaining activity of FAS at different time intervals to obtain the time-course curves. The control is treated with the same procedure without α-mangostin.

### Statistical analysis

Obtained results were analyzed by one way ANOVA test (Origin 8.0). *p* values<0.05 were considered statistically significant and *p* values<0.01 were considered highly significant.
